# Epigenetic Regulator KDM4D Restricts Tumorigenesis *via* Modulating SYVN1/HMGB1 Ubiquitination Axis in Esophageal Squamous Cell Carcinoma

**DOI:** 10.3389/fonc.2021.761346

**Published:** 2021-11-08

**Authors:** Wenjian Yao, Jianjun Wang, Li Zhu, Xiangbo Jia, Lei Xu, Xia Tian, Shuai Hu, Sen Wu, Li Wei

**Affiliations:** ^1^ Department of Thoracic Surgery, Henan Provincial People’s Hospital, People’s Hospital of Zhengzhou University, School of Clinical Medicine, Henan University, Zhengzhou, China; ^2^ Department of Thoracic Surgery, Zhengzhou University People’s Hospital, Henan Provincial People’s Hospital, Zhengzhou, China

**Keywords:** KDM4D, HMGB1, ubiquitination, glycyrrhizin, esophageal squamous cell carcinoma

## Abstract

**Background:**

Increasing researches have been reported that epigenetic alterations play critical roles in ESCC development. However, the role of the histone demethylase KDM4D in ESCC tumorigenesis is poorly investigated. This study aims to discover the underlying mechanisms between KDM4D and ESCC progression.

**Methods:**

CCK-8 assays, clone formation assay and soft-agar assays were performed to assess cell proliferation. Transwell assay was utilized to assess cell migration efficiency, while sphere formation assay was used to evaluate the cell self-renewal ability. Bioinformatic analysis was conducted to identify prognostic factors and predict the potential E3 ubiquitin ligases. *In vitro* ubiquitination assay was conducted to confirm the regulations between SYVN1 and HMGB1. The mRNA levels or protein levels of genes were detected by real-time PCR and western blot analysis. *In vivo* tumor xenograft models were used to determine whether the HMGB1 inhibition affected the malignant features of ESCC cells.

**Result:**

Epigenome screening and low-throughput validations highlighted that KDM4D is a tumor suppressor in ESCC. KDM4D expressed lowly in tumors that predicts poor prognosis. KDM4D deficiency significantly enhanced tumor growth, migration and stemness. Mechanistically, KDM4D transcriptionally activates SYVN1 expressions *via* H3K9me3 demethylation at the promoter region, thereby triggering the ubiquitin-dependent degradation of HMGB1. Low KDM4D depended on accumulated HMGB1 to drive ESCC progression and aggressiveness. Targeting HMGB1 (Glycyrrhizin) could remarkably suppress ESCC tumor growth *in vitro* and *in vivo*, especially in KDM4D-deficient cells.

**Conclusions:**

We systematically identified KDM4D/SYVN1/HMGB1 axis in ESCC progression, proving novel biomarkers and potential therapeutic targets.

## Introduction

Esophageal cancer (EC), ranking sixth in cancer-related causes of death worldwide, remains a common malignant tumor of the digestive tract with poor prognosis (1–3). According to the recent statistics, the estimated new cancer cases derived from esophagus would be 19,260 in 2021, leading to about 15,530 deaths in the USA (4). According to the histological features, esophageal cancer contains esophageal adenocarcinoma (EAC) and squamous cell carcinoma (ESCC), which accounts for ~80% of all diagnosed EC patients (5, 6). The combination strategies, including surgical resection, radiotherapy and chemotherapy, indeed improved the overall survival rate of ESCC (7–9). However, many patients may have no chances to have the surgical treatment for the advanced stages. In recent years, the developed systemic therapies have improved the outcomes, but the 5-year overall survival (OS) rate of ESCC still remains at 15%-25%, caused by distal metastasis or resistance to chemotherapy like cisplatin (10–12). As a result, there is an urgent need for exploring predictive biomarkers and effective therapeutic treatments to treat ESCC.

In recent years, numerous studies have implicated that epigenetic alterations exert essential roles in a wide range of malignancies (13). As reported, DNA methyltransferases, histone modifying enzymes, methyl-DNA binding proteins and non-coding RNAs have been demonstrated to be potential therapeutic targets (14–16). The Jumonji C Domain (JMJD) family has nearly 30 proteins, and 18 of them possess the demethylating enzyme activity (17). Based on the degree of homology and the characteristics of other domains, JMJD proteins are divided into the subfamilies. Among them, JMJD2A–C, also named as lysine (K)-specific demethylase 4 (KDM4), contains double PHD and Tudor domains, which are indicated in specifically binding to methylated histones (18). However, KDM4D is distinct from the other three members in that it possesses neither PHD nor Tudor domains. Previous studies have revealed that KDM4D is indispensable for phosphorylation of a subset of ATM substrates, participating in epigenetic regulation, genome stability and DNA repair responses (19, 20). However, little was reported about the underling mechanisms between KDM4D and the ESCC tumorigenesis.

The high mobility group box 1 (HMGB1) was found in calf thymus and named for its electrophoretic mobility in polyacrylamide gels. Previous studies have implicated that HMGB1 is a chromatin-binding nuclear factor that has been implicated in both infectious and sterile inflammatory disease states, including tumors (21–23). HMGB1 is found to be highly expressed in many solid tumors and elevated HMGB1 could favor a chronic inflammatory state *via* the induction of multiple inflammatory mediators (24). In the cell nucleus, HMGB1 plays a significant role in regulating transcription, DNA replication, oxidative response, or genetic stability (25). Meanwhile, cytoplasmic HMGB1 participates in immune processes by enhancing autophagy, inhibiting apoptosis, or regulating mitochondrial function (26). Rui Kang et al. found that intracellular HMGB1 could suppress oncogenic Kras-driven pancreatic tumorigenesis by restricting chromosome instability-mediated pro-inflammatory nucleosome release, indicating its novel tumor suppressing roles in pancreatic cancer (27). However, higher HMGB1 expression levels facilitate fibroblast activation by RAGE/aerobic glycolysis, driving breast cancer progression and distal metastasis (28). Given the double functions of HMGB1 in modulating inflammation and cancer, we are still uncertain about the underlying relationships between dysregulation of HMGB1 with ESCC.

In the current study, we screened the prognostic epigenetic regulators in ESCC and validated KDM4D as an essential hit. KDM4D expressed lowly in ESCC and targeting KDM4D enhanced ESCC malignant features. We identified the KDM4D/SYVN1/HMGB1 axis in ESCC and proposed that KDM4D may be a novel predictive biomarker and therapeutic target for ESCC progression.

## Methods and Materials

### Cell Lines and Cell Culture Conditions

Human esophageal squamous cell carcinoma cell lines (KYSE30, EC109 and KYSE150) and 293 T cells were were purchased from the China Center for Type Culture Collection (Wuhan, China), which were maintained in RPMI/1640 medium (Gibco) supplemented with 10% fetal bovine serum (Hyclone). Cells were cultured in an atmosphere of 5% CO_2_ at 37°C.

### Cell Proliferation and Colony Formation Assays

Cell proliferation rates were evaluated by CCK-8 assay and colony formation assay. Briefly, we seeded the cells into 96-well culture plates (Corning Life Science). During the timepoint of 24, 48, 72, 96 and 120 h post seeding, the 10 μl CCK-8 reagent was added to the wells and incubated for 1 h. The SpectraMax 190 Microplate Reader (Molecular Devices) was used to assess the absorbance at 450 nm. For the colony formation assay, 1000–2000 indicated cells were seeded into the six-well plates. After two weeks, these wells were fixed with 1.5% crystal violet/methanol solution and counted. Lastly, for the soft-agar colony formation assay, 2000 evaluated KYSE30 cells were mixed into 0.36% agarose culture medium as the upper layer, whereas the bottom layer is mixed with 0.75% agarose and DMEM culture medium. The colony were stained *via* crystal violet and the clone numbers were counted.

### *In Vitro* Transwell Assays

ESAC cells (KYSE30 and EC109) were seeded on the coated filter in 100 μl of serum-free medium, and the bottom chamber was filled with 600 μl complete culture medium. Then, the invasion assays were conducted in the 24-well cell chamber coated with the 30 μl of Matrigel (Corning Incorporated). The invasive ESCA cells were then stained with the crystal violent after 24 h incubation at 37°C under the 5% CO_2_. The migration assays were performed in a similar process without coating with Matrigel.

### *CRISPR/Cas9-*Mediated KDM4D Knock Out Stable Cell Generation

The pX459 plasmid was used to clone guide oligos targeting KDM4D. KYSE30 and EC109 cells were plated and transfected with pX459 constructs overnight. After 24h transfection, 1μg/ml puromycin was added into the culture to screen cells for 72 hours. Afterwards, living cells were seeded into the 96 well plate by limited dilution to isolate monoclonal cell line. The KDM4D knock out cell clones are screened by western blot and confirmed by sanger sequencing. Sequences of specific sgRNAs for targeting KDM4D are listed as the following: sgKDM4D#1: F: 5’-CACCGCCACGCCGATCTGCAGTTAG-3’; R: 5’-AAACCTAACTGCAGATCGGCGTGGC-3’. sgKDM4D#2: F: 5’-CACCGCGACCACGTCTGCCATTTGA-3’; R: 5’-AAACTCAAATGGCAGACGTGGTCGC-3’.

### Collection of Clinical Tumor Samples

A total of 150 pairs primary ESCA tumor tissues and matched normal tissues were collected from patients who received surgical treatment at department of Thoracic Surgery, Henan Provincial People’s Hospital between July 2015 and February 2019. All patients were diagnosed by original histopathological detection. Samples of the collected tissues were preserved in liquid nitrogen and clinical information of patients were also collected. The collection of clinical specimens was approved by the ethics review committee of the Henan Provincial People’s Hospital, and every patient was informed of their consent.

### Immunohistochemistry Analysis

To investigate the presence of KDM4D, HMGB1 and Ki67 proteins in tumor tissues or mice, the IHC assay was performed. The following antibodies were used in this assay: KDM4D with 1:4000 dilution; HMGB1 with 1:100 dilution. The human ESCA tissues and matched adjacent tissues used in this study were obtained from Henan Provincial People’s Hospital. These individuals did not receive any radiotherapy, chemotherapy or immunotherapy before surgery and were informed consent.

### *In Vitro* Ubiquitination Assay

Flag-HMGB1 together with HA-Ub and Myc-SYVN1 plasmids were transfected into HEK293T, respectively. Ubiquitinated HMGB1 proteins were immunoprecipitated by anti-Flag affinity gel with an incubation of 10 μM MG132 for 6 h. Purified HMGB1 were obtained after elution with FLAG or MYC peptides and dialysis. They were incubated in a deubiquitination reaction buffer at 30°C for 2 h and analyzed by immunoblotting as previously described.

### Animal Experiments

Eighteen 6 weeks old male BALB/c-nude mice were randomly divided into three groups, and subcutaneously injected with 6×10^5^ cancer cells into the right flank region of each mouse. After visible tumors had developed, tumor volume was measured and calculated as length × width2/2 every 2 days. The Institutional Animal Care and Use Committee of Chinese Academy of Henan Provincial People’s Hospital has approved these experiment procedures.

### Statistical Analysis

The two-tailed Student’s t-test was used to compare results from two groups. The *Pearson* correlation analysis was conducted to determine the correlation between two groups. Results from *in vitro* assays exhibited in the study are representative of three independent experiments. All quantitative data are indicated as Mean ± SD. GraphPad Prism Version 6.01 was utilized for statistical analysis. A *P*-value < 0.05 was considered to be statistically significant.

## Results

### Epigenome Screening Identifies KDM4D as an Epigenetic Suppressor in Esophageal Squamous Cell Carcinoma

**Figure 1:**
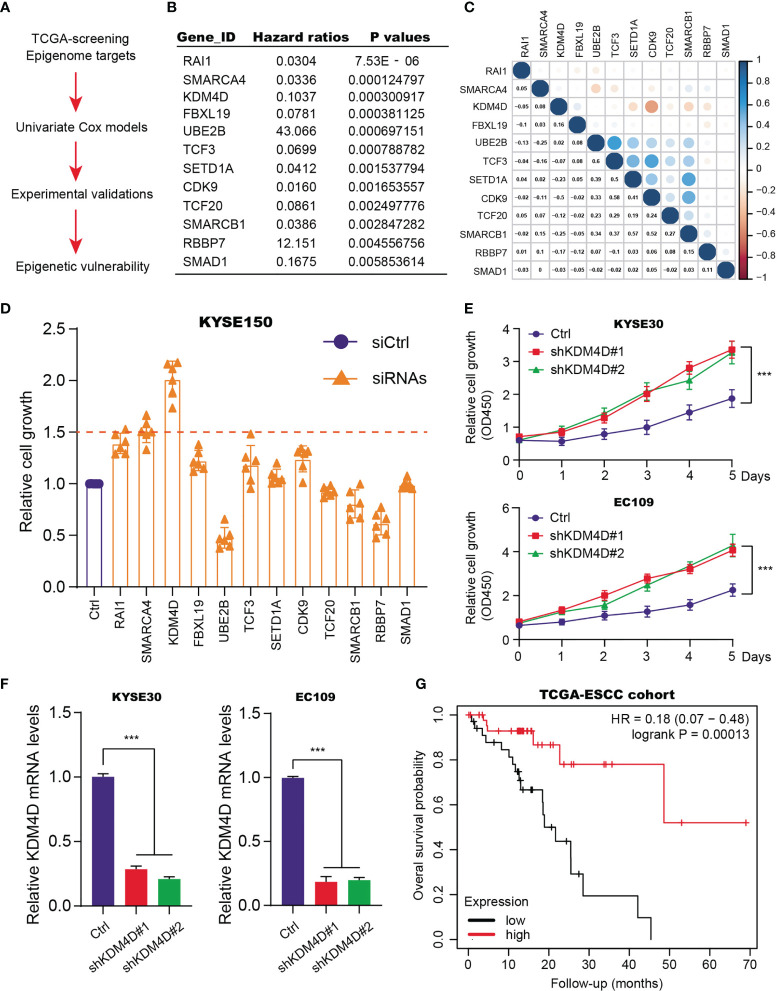
Identification of KDM4D as a prognostic factor for ESCC. **(A)** Screening procedure of epigenetic factors with prognostic significance in ESCC. **(B)** Exhibition of partial results of epigenetic factors based on univariate Cox regression analysis. **(C)** The correlation heatmap revealing the underlying associations across indicated candicates. **(D)** MTT assays in KYSE150 identify that KDM4D was the most potent hit. **(E)** Knockdown assays with specific shRNAs in the KYSE30 and EC109 cell lines. **(F)** Detection of KDM4D mRNA levels *via* qPCR in cells transfected by shCtrl and shKDM4D#1 or shKDM4D#2. **(G)** Kaplan-Meier analysis suggesting the prognostic role of KDM4D in ESCC. *P < 0.05, **P < 0.01, ***P < 0.001.

Given the essential roles of epigenetic remodeling in ESCC tumorigenesis, we intended to screen potential chromatin regulators that are indispensable for tumor progression. Firstly, we extracted the expression data of 562 epigenetic genes from the TCGA-ESCC cohort. The corresponding clinical characteristics of patients, like age, gender, pathological stages, TNM stages, were all collected and summarized in **Table S1**. The univariate Cox regression analysis was conducted to screen 20 prognostic candidates with *P* < 0.01 as the cutoff (**Figures 1A, B** and **Table S2**). Intriguingly, we found several oncogenic targets associated with ESCC, like UBE2B, TCF20 and RBBP7. Meanwhile, in line with previous studies, KDM4D, SMARCA4 and RAI1 were shown to be suppressors in ESCC. Besides, the underlying associations among the 12 essential epigenetic candidates were calculated and illustrated in the correlation heatmap (**Figure 1C**). To further identify the hub targets, we selected the top 12 candidates and designed specific siRNAs to perform a validation screen. As shown by MTT assay in KYSE150, KDM4D was the most potent hit (**Figure 1D**). Though targeting RAI1, SMARCA4 or KDM4D indeed resulted in elevated cell growth, KDM4D inhibition led to the most increase of growth rates compared with other candidates. In addition, we further used 2 different shRNA constructs to deplete KDM4D expression and found that reduced KDM4D expression significantly enhanced the growth of ESCC cells, including KYSE30 and EC109 (**Figure 1E**). The knockdown efficiency of KDM4D in two cell lines were confirmed by qPCR assays (**Figure 1F**). We collected the expression data of KDM4D and related survival data of patients. Kaplan-Meier analysis revealed that ESCC patients with high KDM4D had worse overall survival (OS) outcomes relative to those with low KDM4D levels (**Figure 1G**).

### KDM4D Expresses Lowly in ESCC That Predicts Poor Prognosis

**Figure 2:**
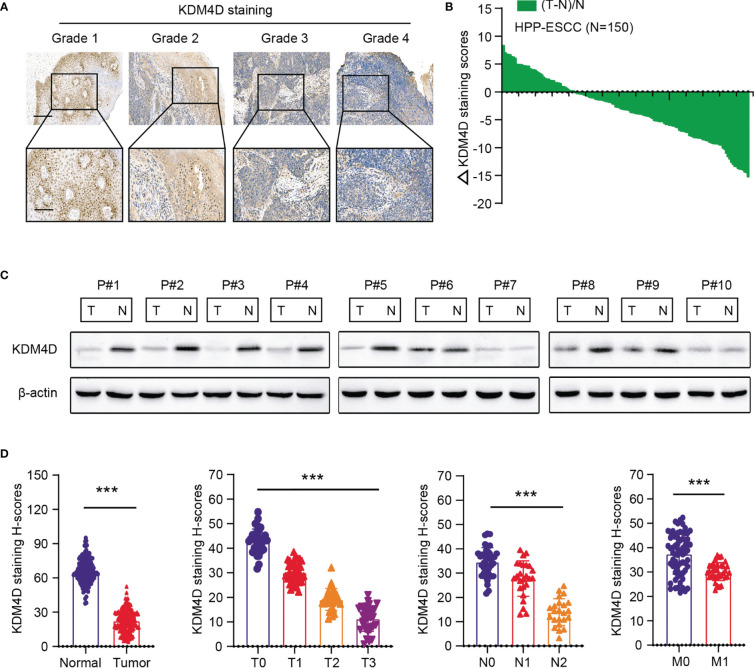
Validation of expression levels and clinical significance of KDM4D in ESCA. **(A)** Representative IHC graphs in ESCC samples with different grades. **(B)** Quantification of the H-scores of KDM4D staining in HPP-ESCC cohort. Scale bar = 200μm (upper) and Scale bar = 80μm (lower). **(C)** KDM4D protein levels were detected in ESCC tissues and paired normal tissues *via* western blotting (n = 10). **(D)** Correlation analysis of KDM4D protein levels with clinical characteristics in HPP-ESCC cohort. *P < 0.05, **P < 0.01, ***P < 0.001.

Accordingly, we collected tumors from totally 150 patients from the Henan Provincial People’s Hospital to determine the expression levels of KDM4D in ESCC. First of all, we found KDM4D expressed lowly in tumor samples relative to normal tissues (**Figure 2A**). Besides, low KDM4D correlated with advanced tumor grades, as evidenced by the IHC graphs (**Figure 2A**). In addition, we quantified the H-scores of KDM4D staining and calculated the ΔKDM4D across samples (**Figure 2B**). In line with these findings, the protein levels of KDM4D were further found to be significantly lower in 7/10 (70%) human ESCC tissues than in their paired normal tissues through western blot assays (**Figure 2C**). Lastly, we integrated the KDM4D expression data with clinical information and confirmed that KDM4D correlated negatively with hazard factors, including T stages, N stages and tumor grades (**Figure 2D**). Collectively, these data implicated that KDM4D expressed lowly in ESCC and correlated with hazard clinical parameters.

### Targeting KDM4D Enhances Tumor Progression and Chemotherapy Resistance

**Figure 3:**
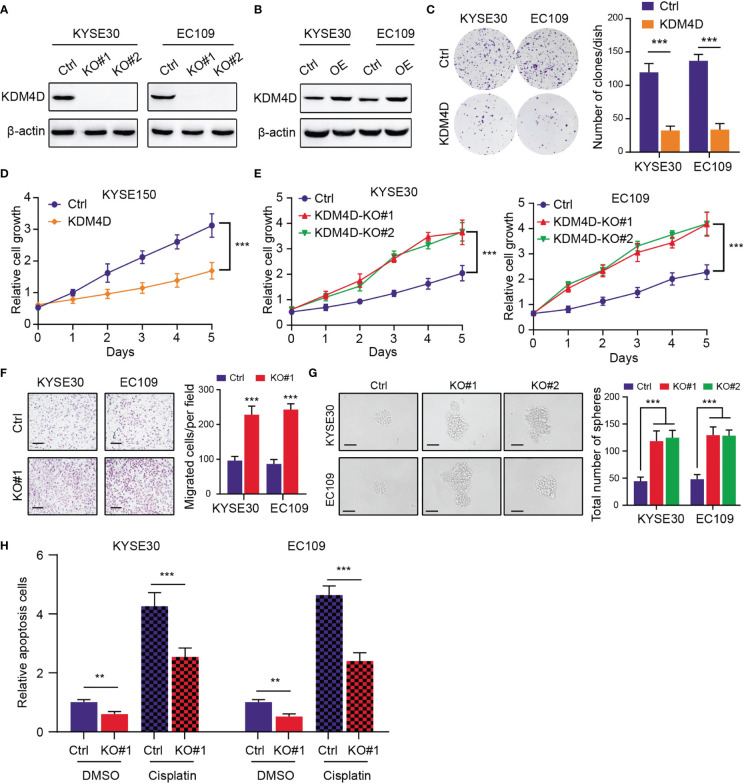
KDM4D inhibition promotes tumor progression and chemotherapy resistance. **(A)** Western blot revealing the knockout efficiency of KDM4D inKYSE30 and EC109. **(B)** Western blot exhibiting the overexpression of KDM4D in KYSE30 and EC109. **(C)** Overexpression of KDM4D suppressed the colony-formation ability of ESCC cells (left panel). Quantification of the colony formation assay results (right panel). **(D)** CCK-8 assays showing the difference of growth rate in Ctrl and KDM4D-overexpressing cells. **(E)** CCK-8 assays comparing the growth differences in Ctrl and KDM4D-KO cells. **(F)** KDM4D deficiency promoted the migrated ability of KYSE60 and EC109 cells (left panel). Quantification of the Transwel assay results (right panel). **(G)** Knockout of KDM4D induced the increase in sphere numbers and sizes of ESCC (KYSE30 and EC109) cells relative to the corresponding control cells (left panel); Quantification of results from the sphere formation assays. **(H)** Apoptosis rates were detected in cells treated with DMSO and cisplatin. *P < 0.05, **P < 0.01, ***P < 0.001.

To further elucidate the functional roles of KDM4D in ESCC, we utilized the *CRISRP/Cas9* technology to delete KDM4D in ESCC cells (KYSE30 and EC109), which was validated *via* western blot (**Figure 3A**). Besides, we utilized the lentivirus-mediated transfection to construct the KMD4D-overexpressing ESCC cells (KYSE30 and EC109), which was confirmed *via* western blot (**Figure 3B**). The up-regulation of KDM4D notably suppressed the clonogenic ability of ESCC cells (KYSE30 and EC109), as indicated by clone numbers (**Figure 3C**). Meanwhile, CCK-8 assays revealed that KDM4D overexpression attenuated the growth effect of cells (KYSE150) (**Figure 3D**), whereas KDM4D knockout significantly enhanced the growth of ESCC cells (KYSE30 and EC109) (**Figure 3E**). To determine the roles of KDM4D in ESCC metastasis, we observed that inhibition of KDM4D promoted the migration ability of KYSE30 and EC109 cells (**Figure 3F**). Interestingly, an increase in sphere numbers and sizes was observed in KDM4D-deficient KYSE30 and EC109 cells compared with the corresponding control cells, indicating that down-regulation of KDM4D could enhance ESCC self-renewal ability (**Figure 3G**). Considering that tumor stemness correlated tightly with drug resistance, we treated the ESCA cells with cisplatin and observed that KDM4D loss rendered cells more resistant to chemotherapy-induced apoptosis (**Figure 3H**). Taken together, our data indicated that KDM4D is a tumor suppressor and KDM4D inhibition could promote ESCC proliferation, migration, stemness features and resistance to chemotherapy.

### Low KDM4D Depends on Accumulated HMGB1 to Potentiate Tumor Malignant Features

**Figure 4:**
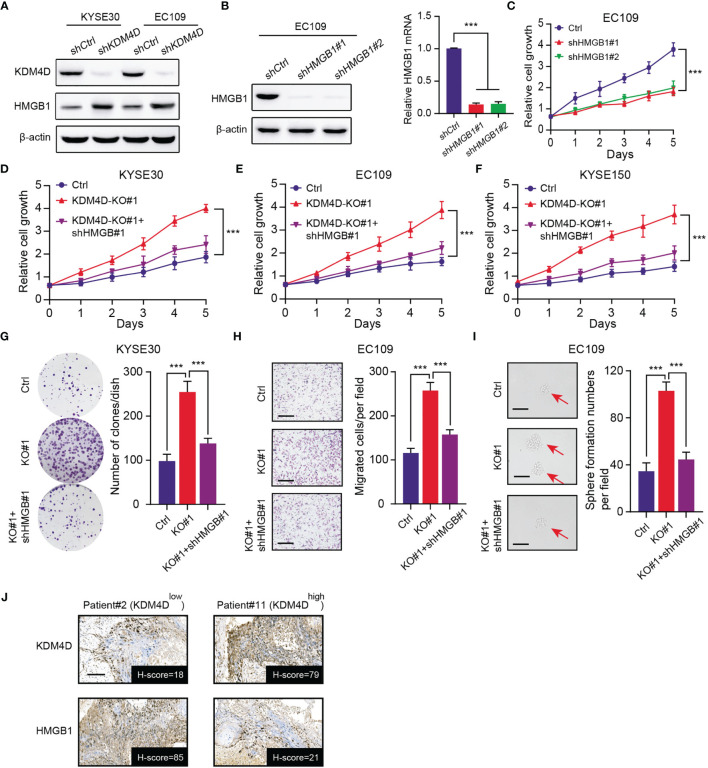
Low KDM4D depends on elevated HMGB1 to promote ESCC progression. **(A)** Western blot assays detecting the protein levels of KDM4D and HMGB1 in KYSE30 and EC109 cells. **(B)** Western blot exhibiting the knockdown efficiency of KDM4D by specific shRNAs in EC109 cells (left). qPCR assay revealing the mRNA levels of HMGB1 (right). **(C)** CCK-8 assays revealing the cell growth differences in Ctrl and HMGB1-knockdown EC109 cells. **(D–F)** HMGB1 knockdown significantly suppressed the cell growth in KDM4D-deficient cells (KYSE30, EC109 and KYSE150), as indicated by the CCK-8 assays. **(G)** HMGB1 inhibition significantly impaired the clone formation efficiency in KDM4D-deficient KYSE30 cells. **(H)** HMGB1 inhibition significantly impaired the migration efficiency in KDM4D-deficient EC109 cells. **(I)** HMGB1 inhibition significantly impaired the self-renewal ability in KDM4D-deficient EC109 cells. **(J)** Representative images showing the negative associations of KDM4D and HMGB1 protein levels. Scale bar = 80μm. *P < 0.05, **P < 0.01, ***P < 0.001.

Our previous data implicated that HMGB1 proteins were up-regulated in KDM4D-knockdown cells (KYSE30 and EC109) (**Figure 4A**). As reported, HMGB1 is an onco-protein that plays essential roles in tumorigenesis and stemness processes across multiple tumors, including ESCC. We designed specific shRNAs to knockdown HMGB1 in ESCC cells, which was validated by western blot and qPCR assays (**Figure 4B**). As expected, targeting HMGB1 indeed remarkably suppressed cell growth, as evidenced by the CCK-8 assay (**Figure 4C**). Besides, we inhibited HMGB1 by specific shRNAs in KDM4D-deficienct ESCC to conduct the CCK-8 assays. In line with our speculations, KDM4D deficiency could elevated cell growth rates (KYSE30, EC109 and KYSE150), which could be significantly suppressed when HMGB1 was down-regulated (**Figures 4D–F**). The clone formation efficiency of KDM4D-deficient ESCC could also be notably impaired with HMGB1 inhibition (**Figure 4G**). Similarly, we also conducted the migration assay and confirmed that HMGB1 knockdown could also suppressed the migration ability induced by KDM4D deficiency (**Figure 4H**). Lastly, we also found that KDM4D deficiency depended on HMGB1 to enhance stemness potentiality of EC109 cells. (**Figure 4I**). We further collected the ESCC samples and found the negative expression levels between KDM4D and HMGB1 *via* IHC assays (**Figure 4J**). Taken together, our findings implicated that low KDM4D depended on elevated HMGB1 to drive ESCC malignant features, including proliferation, migration or self-renewal abilities.

### KDM4D Activates SYVN1 to Promote HMGB1 Degradation and Restrict Its Protein Levels

**Figure 5:**
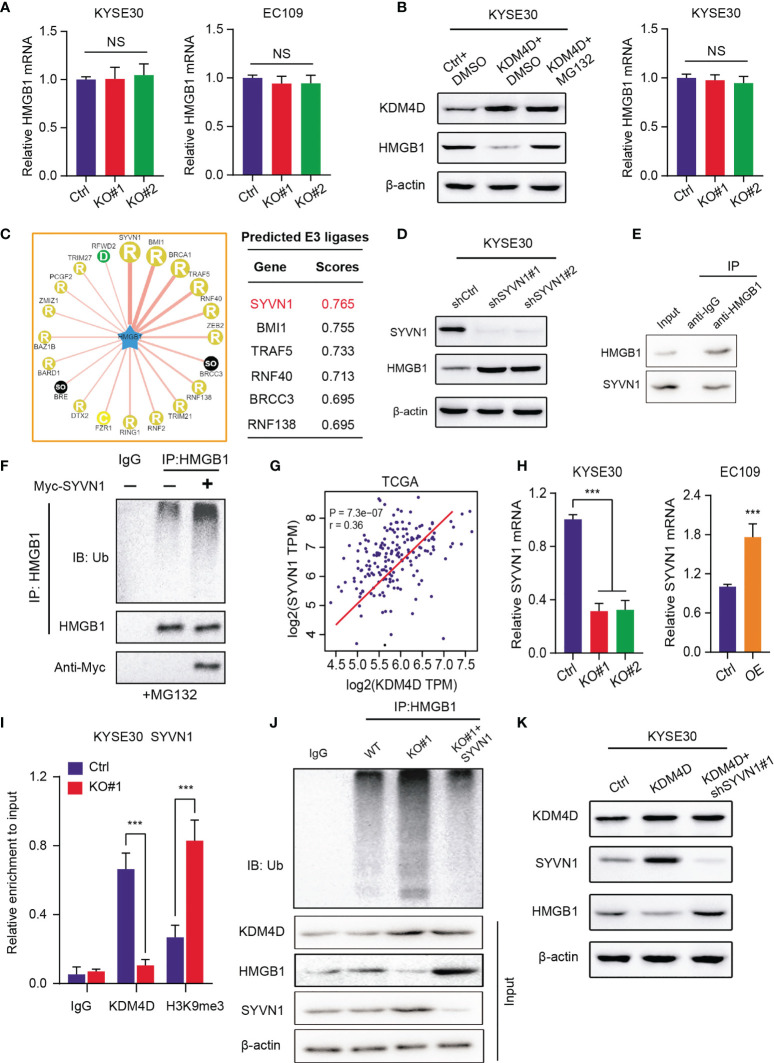
KDM4D activates SYVN1 to promote HMGB1 degradation and restrict its protein levels. **(A)** Detection of HMGB1 mRNAs in Ctrl and KDM4D-KO cells (KYSE30 and EC109). **(B)** Detection of protein levels and mRNA levels of HMGB1 in three groups, including Ctrl+DMSO, KDM4D+DMSO and KDM4D+MG132. **(C)** Bioinformatic analysis for predicting E3 ubiquitin ligases that target HMGB1 for degradation based on the UbiBrowser dataset. **(D)** Western blot assays revealing the associations between SYVN1 and HMGB1. **(E)** Co-IP assay detecting the endogenous interactions between SYVN1 and HMGB1. **(F)** Western blot of the products of *in vitro* ubiquitination assays from 293 T cells transfected with the indicated plasmids and treated with 20 μM MG132 for 8 h. **(G)** Correlation analysis revealing the associations between KDM4D and SYVN1 mRNA levels in TCGA-ESCC cohort. **(H)** Detection of SYVN1 mRNA levels in different groups, as indicated. **(I)** The ChIP-PCR was conducted to confirm the occupancy of KDM4D and H3K9me3 modification at the SYVN1 promoter in KYSE30 cells. **(J)** Western blot of the products of *in vitro* ubiquitination assays from 293 T cells transfected with the indicated plasmids and treated with 20 μM MG132 for 8 h. **(K)** Western blot assays revealing the associations across KDM4D, STVN1 and HMGB1 in three groups, including Ctrl, KDM4D and KDM4D+shSYVN1#1. ***P < 0.001. NS, No Significance.

To elucidated the underlying mechanisms that KDM4D relies on to modulate HMGB1, we first detected the mRNA levels of HMGB1 in ESCC cells. Intriguingly, we found that KDM4D deficiency promoted the elevated HMGB1 protein levels. However, no alterations of mRNA levels of HMGB1 were detected in KDM4D-KO and control cells (**Figure 5A**). Of note, MG132, one well-known proteasome inhibitor, could significantly restored the protein levels of HMGB1 in KDM4D-deficient cells, but not the mRNA levels (**Figure 5B**). Briefly, we speculated that KDM4D may regulate HMGB1 proteins at the post-transcriptional level, especially through the ubiquitin-proteasome pathway. Given that KDM4D is a chromatin regulator, not the direct ubquitin ligase, we thus considered there might be other ligases that linked KDM4D and HMGB1. We queried the UbiBrowser website (http://ubibrowser.ncpsb.org.cn/ubibrowser/) to predict the essential E3 ubiquitin ligases that target HMGB1 (**Figure 5C**). After validations, we found that only SYVN1 inhibition could increase HMGB1 levels (**Figure 5D**). The endogenous interactions between HMGB1 and SYVN1 were confirmed by the Co-immunoprecipitation (Co-IP) analysis using the anti-HMGB1 antibody (**Figure 5E**). Importantly, SYVN1 mediated the polyubiquitination process of HMGB1, indicating that HMGB1 is the SYVN1 downstream substrate (**Figure 5F**). SYVN1 inhibition remarkably prolonged the half-life of HMGB1 protein in EC109 cells (**Figure S1A**). Meanwhile, positive associations between KDM4D and SYVN1 were confirmed in TCGA-ESCC cohort (r=0.36, *P*<0.0001, **Figure 5G**). KDM4D loss could notably decrease SYVN1 mRNA levels, and KDM4D overexpression could elevate its mRNA levels relative to control cells (**Figure 5H**). Previous data implicated that KDM4D transcriptionally activates downstream targets *via* H3K9me3 demethylation at the promoter region (29, 30). ChIP-qPCR assay was performed to confirm that KDM4D deficiency could indeed increase the H3K9me3 binding abilities at the promoter of SYVN1 (**Figure 5I**). Additionally, KDM4D overexpression indeed enhanced the polyubiquitination levels of HMGB1, which could be notably impaired upon knockdown of SYVN1 (**Figure 5J**). Lastly, KDM4D overexpression decreased the HMGB1 protein levels, but SYVN1 inhibition restored the corresponding proteins (**Figure 5K**). Collectively, our findings indicated that KDM4D restricts HMGB1 protein levels *via* activating SYVN1 expressions.

### HMGB1 Inhibitor (Glycyrrhizin) Effectively Suppresses *In Vitro* and *In Vivo* Tumor Growth of KMD4D^low^ ESCC

**Figure 6:**
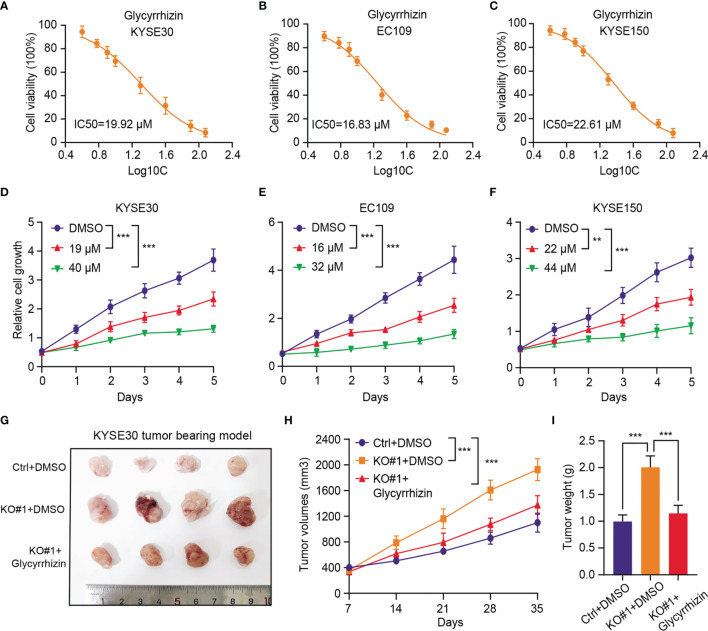
HMGB1 inhibitor (Glycyrrhizin) notably suppresses *in vitro* and *in vivo* tumor growth of KMD4D^low^ ESCC. **(A-C)** Detection of the half maximal inhibitory concentration (IC50) values of Glycyrrhizin in three ESCC cell lines (KYSE30, EC109 and KYSE150). **(D-F)** The cell growth ability of the ESCC cells (KYSE30, EC109 and KYSE150) were all remarkably impaired with Glycyrrhizin treatment in a dose‐dependent manner. **(G)** HMGB1 inhibition (Glycyrrhizin) effectively suppressed ESCC subcutaneous tumour growth in nude mice (n = 5). **(H)** The tumour volume was detected every other day, and tumour growth curves were generated. **(I)** The tumours were extracted and weighed after 35 days. *P < 0.05, **P < 0.01, ***P < 0.001.

Considering that KDM4D is an essential molecular biomarker in ESCC and no selective inhibitors are available, we thus wondered whether HMGB1 inhibitor could be effective to treat ESCC, especially the KDM4D^low^ tumors. Glycyrrhizin, a major constituent of licorice root, is a highly selective drug that targets HMGB1. The half maximal inhibitory concentration (IC50) values of Glycyrrhizin were first evaluated and calculated in three ESCC cell lines (**Figures 6A–C**). Then, we found that the cell growth ability of the ESCC cells (KYSE30, EC109 and KYSE150) were all remarkably impaired with Glycyrrhizin treatment in a dose‐dependent manner (**Figures 6D–F**). In line with the *in vitro* results, we also observed that Glycyrrhizin could effectively suppressed the elevated *in vivo* tumor growth induced by KDM4D deficiency, as indicated by the tumor volumes and weight (**Figures 6G–I**). Cumulatively, these results established that targeting HMGB1 (Glycyrrhizin) is proved to be an effective strategy to inhibit ESCC tumor growth, especially for the KDM4D^low^ cases.

## Discussion

Although there are currently developed and combined strategies to treat ESCC, the overall prognosis of ESCC still remains unsatisfactory. The tumorigenesis and progression of ESCC correlate with multiple mechanisms and biological processes, including dysregulation of tumor immune microenvironment, activation of kinases signaling, as well as metabolic disorders (31–33). Among them, epigenetic remodeling has been well documented and considered to be essential molecular features of tumorigenesis (34, 35). Here, we screened the prognostic epigenetic regulators in TCGA-ESCC and identified KDM4D as a tumor suppressor *via* low-throughput validations. KDM4D expressed lowly in tumor samples relative to normal tissues and low KDM4D correlated with hazard clinical characteristics and poor survival outcomes. KDM4D inhibition could enhance ESCC cells proliferation, migration and self-renewal abilities, depending on accumulated HMGB1 protein levels. Based on predictive analysis, we identified SYVN1 could promote the HMGB1 polyubiquitination. KDM4D mediated the demethylation of H3K9me3 at the promoter region of SYVN1 to activate it expressions. KDM4D thus could elevate SYVN1 transcriptional levels to enhance HMGB1 degradation. We also demonstrated that HMGB1 inhibitor (Glycyrrhizin) is effective in suppressing ESCC growth *in vitro* and *in vivo*.

Previous studies have already found that KDM4D may function as an oncogene that modulates multiple cellular processes, including transcriptional regulation, cell differentiation, stem cell renewal, and tumor metastasis (18, 19, 36). Compared with other histone methylase or demethylase, KDM4D is the most abundant histone demethylase that facilitate the decrease of H3K9me2 and H3K9me3 level during the processes of hepatic stellate cells (HSCs) activation (30). Accordingly, Fuqing Hu et al. observed that HIF1β transcription activation by KDM4D is highly correlated with H3K9me3 and H3K36me3 demethylation (29). Overexpressed KDM4D in gastrointestinal stromal tumor depended on HIF1β to promote VEGFA signaling pathway. Similarly, KDM4D and β-catenin interacted physically and KDM4D demethylated H3K9me3 at promoters of β-catenin downstream genes (37). In line with these findings, we observed that KDM4D binds at the SYVN1 promoter region and promotes the H3K9me3 demethylation, leading to high expression levels of SYVN1. However, KDM4D mainly exerted the suppressive roles in ESCC to restrict cell growth and migration. Cancer stem cells (CSCs) originate from tumor cells that possess notable properties of self-renewal, clonal tumor initiation capacity (38, 39). Tumor stemness features are tightly associated with tumor metastasis, distal recurrence and chemotherapy resistance (40). Functional experiments implicated that low KDM4D ESCC tumors depended on HMGB1 to potentiate the tumor stemness. Given that enhanced tumor stemness could largely contribute to chemotherapy resistance, we accordingly detected that KDM4D^low^ ESCC were highly resistant to cisplatin-induced apoptosis compared with controls (41). We therefore proposed that KDM4D might also function as an effective biomarker for stratification of cisplatin treatment in ESCC. Meanwhile, whether there existed other downstream targets of KDM4D, like NF-κB signaling or Hedgehog crosstalk, that contribute to ESCC progression and stemness remains to be further elucidated.

(42) In eukaryotes, the ubiquitin-proteasome system (UPS) contains ubiquitin-activating enzyme (E1), ubiquitin-conjugating enzyme (E2s), ubiquitin ligase (E3s), and proteasome (43). Aberrant ubiquitination-mediated degradation of hub proteins contribute to tumor progression, metastasis and therapy resistance in multiple tumors, including ESCC (44). EIF3H is significantly upregulated in esophageal cancer and functions as a novel deubiquitinating enzyme of Snail, driving aggressiveness and metastasis (45). Sisi Wei et al. found that overexpression of SNHG5 decreased the transcription of MTA2 and resulted in its ubiquitin-mediated degradation in esophageal cancer (46). Previous studies have suggested that HMGB1 functions as an oncogene in various tumors and dysregulation of HMGB1 is well investigated and documented. As reported, HSF1 is upregulated in OVA-induced asthmatic mice and directly binds with the HMGB1 promoter and negatively regulation of HMGB1 (47). Yao Liu et al. reported that hypoxia condition could promote HMGB1 to translocate from the nucleus to the cytosol and bind to mtDNA released from damaged mitochondria, activating TLR9 signaling and tumor growth of hepatocellular carcinoma (26). In this study, we identified that KDM4D/SYVN1 axis could modulate the post-transcriptional regulation of HMGB1, providing novel insights on HMGB1 accumulations in tumor. Previous studies have suggested that E3 ubiquitin-protein ligase SYVN1 expressed highly in liver cancer and interacted with HSP90 to drive metastasis and vascular invasion (48). However, our study proposed that SYVN1 may be a tumor suppressor that target HMGB1 for degradation. Whether SYVN1 exerts other biological effects remains interesting to be discovered.

However, this study still has several defects and problems that need to be improved and discovered further. First of all, we proposed that KDM4D may be a predictive biomarker in ESCC. However, the cutoff that discriminates high- and low-KDM4D cases is still indefinite. More ESCC samples with IHC staing from multiple centers are warranted to define the relatively clear threshold for KDM4D. Besides, Glycyrrhizin is proved to be effective in ESCC to inhibit tumor growth. Given that little was reported about the use of Glycyrrhizin in ESCC, the specific side effects should be well investigated and evaluated. Meanwhile, patient-derived tumor xenograft (PDX) or patient-derived organoids (PDOs) were all recommended to detect the clinical significance of Glycyrrhizin in ESCC. Last but not least, we discovered that KDM4D/SYVN1/HMGB1 axis plays essential roles in stemness features and apoptosis resistance. We thus wondered that whether Glycyrrhizin treatment could abrogate the chemotherapy resistance and have synergistic effect with cisplatin in KDM4D^low^ tumors.

## Conclusion

Taken together, our findings revealed a suppressive role of KDM4D in ESCC development. Mechanistically, KDM4D/SYVN1/HMGB1 axis modulates the tumor growth, migration and self-renewal features *via* promoting accumulations of HMGB1. Moreover, KDM4D/HMGB1 could be effective biomarkers for chemotherapy treatment. As a result, epigenetic regulator KDM4D might be a potential predictor and therapeutic indicator for ESCC.

## Data Availability Statement

The original contributions presented in the study are included in the article/**Supplementary Material**. Further inquiries can be directed to the corresponding authors.

## Ethics Statement

The studies involving human participants were reviewed and approved by the Ethics committee of Henan Provincial People’s Hospital, Zhengzhou. The patients/participants provided their written informed consent to participate in this study. The animal study was reviewed and approved by The Institutional Animal Care and Use Committee of Chinese Academy of Henan Provincial People’s Hospital. Written informed consent was obtained from the owners for the participation of their animals in this study.

## Author Contributions

SW and LW conceived the concept of the study. WY and JW conducted the experimental assays. LZ, XJ and LX collected the ESCA samples and conducted the IHC assays. XT and SH performed the statistical analysis. WY and JW wrote the draft of the study and LW revised the paper. All authors have approved the final version of paper.

## Funding

This study was supported by Key Science and Technology Projects in Henan Province (212102310669, 202102310457), Henan Province medical science and technology research plan joint construction project (LHGJ20200034) & “23456 Talent Project” of Henan Provincial People’s Hospital.

## Conflict of Interest

The authors declare that the research was conducted in the absence of any commercial or financial relationships that could be construed as a potential conflict of interest.

## Publisher’s Note

All claims expressed in this article are solely those of the authors and do not necessarily represent those of their affiliated organizations, or those of the publisher, the editors and the reviewers. Any product that may be evaluated in this article, or claim that may be made by its manufacturer, is not guaranteed or endorsed by the publisher.

## Supplementary Material

Supplementary Figure 1 A Western blot of indicated proteins in WCLs of EC109 cells infected with lentivirus expressing SYVN1-specific shRNA or NC for 48 h and then treated with 50 μg/ml cycloheximide (CHX) and harvested at different time points.

## Abbreviations

ESCC, esophageal squamous cell carcinoma; HMGB1, The high mobility group box 1; TCGA, The Cancer Genome Atlas; CCK8, Cell Counting Kit-8; KDM4D, lysine demethylase 4D; KO, Knockout; WT, Wild type; ChIP, Chromatin Immunoprecipitation; IC50, The half maximal inhibitory concentration.
