# Supplementary figures and images for: Epigenetic Regulator KDM4D Restricts Tumorigenesis via Modulating SYVN1/HMGB1 Ubiquitination Axis in Esophageal Squamous Cell Carcinoma
Source: Front Oncol. 2021 Nov 8;11:761346. doi: 10.3389/fonc.2021.761346 (PMC8606580; doi:10.3389/fonc.2021.761346)

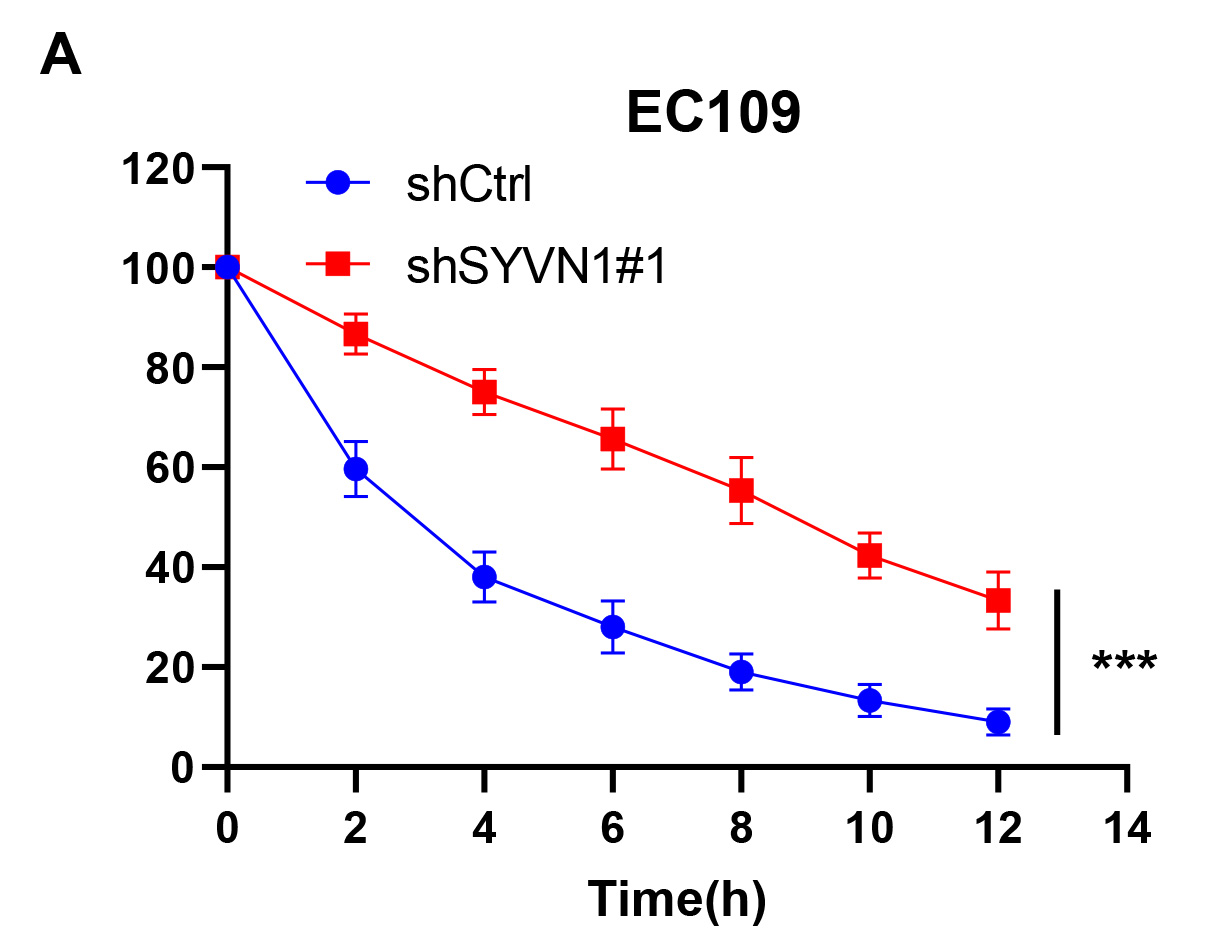

Supplement: Supplementary Figure 1 — A Western blot of indicated proteins in WCLs of EC109 cells infected with lentivirus expressing SYVN1-specific shRNA or NC for 48 h and then treated with 50 μg/ml cycloheximide (CHX) and harvested at different time points. [file Image_1.jpeg]
